# Reasons it is doubtful that preconceptional paternal irradiation with plutonium-239 had any effect on cancer induction by methyl-nitroso-urea

**DOI:** 10.1038/sj.bjc.6690812

**Published:** 1999-11

**Authors:** P B Selby

**Affiliations:** Life Sciences Division, Oak Ridge National Laboratory, 1060 Commerce Park Drive, Oak Ridge, TN, 37830–6480, USA


*British Journal of Cancer* (1999) **81**(6), 1094–1096
© 1999 Cancer Research Campaign
Article no. bjoc.1999.0812

Letters to the Editor

### Reasons it is doubtful that preconceptional paternal irradiation with plutonium-239 had any effect on cancer induction by methyl-nitroso-urea

**Sir**

Recently Lord and colleagues (1998) published a paper in this journal in which they claimed that preconceptional paternal irradiation (PPI) with $^{239}$Pu substantially affected development of haemopoietic stem cells, produced cytogenetic aberrations, and markedly altered and increased cancer induction following intravenous injection of male mice with 50 mg kg$^{-1}$ methyl-nitroso-urea (MNU). They speculated that the cancer effects and the cytogenetic aberrations might result from the effects on haemopoietic stem cells, which had to result from transmitted radiation-induced mutations if PPI was the cause. I will provide some of the reasons why it seems implausible that PPI was responsible for the claimed cancer effects. Indeed, from the way in which the data were presented, it is unclear whether PPI had any effect in the Lord et al study.

The paper presented no dosimetry to estimate the doses in mGy of PPI that resulted from their injections of male mice with either 128 or 256 Bq g$^{-1}$ of $^{239}$Pu-citrate. (These two dose groups will be referred to as the 128-PPI and 256-PPI groups.) Comparisons with extensive studies (NCRP 1981) of the mutational effects of injections of 370 Bq g$^{-1}$ of $^{239}$Pu-citrate at Oak Ridge National Laboratory (ORNL) provide a means for estimating that, at most, the doses in the 128-PPI and 256-PPI groups were 160 and 310 mGy, respectively. Knowledge of these doses is useful for comparisons below.

The following three specific effects on MNU-induced cancer induction were reported to result from PPI (and thus from radiation-induced mutations): markedly reduced time until first neoplasm, considerable shortening of the time required until 50% of the mice developed malignancy, and a pronounced shift from thymic lymphomas to leukaemias. Because of the manner of data presentation, it takes some effort to ascertain the induced frequencies of radiation-induced mutations required to yield the observed effects. The observed mutation frequencies for each dose can be derived by multiplying the percentage of mice developing cancer by day 250 after MNU injection times the fraction of that group said to have leukaemia. Accordingly, for the doses of 0, 160 and 310 mGy, the observed mutation frequencies are 26%, 60% and 53% respectively. Induced mutation frequencies, calculated by subtracting control from experimental, are 34% and 27% in the 160 and 310 mGy groups respectively. It is emphasized that these mutation frequencies are for a very specific type of dominant mutation that causes mice to develop leukaemia (instead of thymic lymphoma) within 250 days after treatment with 50 mg kg$^{-1}$ MNU.

Such high induced mutation frequencies for such a narrowly-defined type of mutation, based on samples of only 62 and 41 offspring respectively, seem extraordinarily improbable if one considers results that have been found following injection of male mice with $^{239}$Pu-citrate for induction of types of gene mutations that have been considered useful for conventional hereditary risk estimation (UNSCEAR, 1993). In 186 275 offspring of male mice that received a mean dose of 1200 mGy of this type of PPI (with a range of 250–2750 mGy), there were 37 specific-locus mutations (NCRP, 1981). Correcting for the spontaneous mutation frequency, this is an induced mutation frequency of about 0.015% for recessive mutations at the combined total of seven genes studied in that method. (On average, those genes are thought to be much more mutable than most mammalian genes.) Because of concern raised by the specific-locus results that some of the induced mutations might have had adverse dominant effects on viability, and because of results showing that similar Pu treatments induce dominant lethal mutations (Lüning et al, 1976), I did a large experiment to investigate the effects of PPI from $^{239}$Pu-citrate in inducing dominant skeletal mutations. The mean dose to the fathers was 580 mGy. The observed frequencies of newly-arising presumed dominant skeletal mutations were six in 3353 offspring in the experimental group and three in 1987 offspring in the concurrent control (Selby, 1990). The point estimate of the induced mutation frequency was 0.03% per gamete, which did not approach statistical significance. This frequency relates to induction of dominant mutations at a presumably vast number of genes capable of causing such skeletal malformations when they do mutate (Selby, 1990).

ORNL specific-locus and heritable translocation results for $^{239}$Pu-citrate have another important bearing on speculation in the Lord et al paper because they provide evidence that the dose–response is linear over the long exposure times involved, with no indication of a humped dose–response that might suggest a higher response with lower doses (NCRP, 1981). While humped dose–responses are well known for doses of radiation that cause extensive spermatogonial killing, there is no basis for invoking that phenomenon for doses as small as those in the Lord et al study. Reports of radiation-induced genetic instability in somatic cells and cells in culture sometimes indicate a much higher response than can be attributed to DNA damage; however, extrapolation of such findings in an attempt to explain the implausibly high induced mutation frequencies in the present study would be unreasonable because DNA changes that result in transmission to offspring are (with rare exceptions not applicable here) only inherited by one animal per mutation.

It seems clear from the above that the observed cancer differences have nothing to do with the injected plutonium-citrate solutions. The true explanation may lie in a poorly defined understanding of the variation in the cancer response from replicate to replicate when such small samples of mice are injected with MNU (without any injection of Pu-citrate). Alternatively, some bias may have inadvertently crept into the experiment. Surprisingly little detail is provided for the cancer part of the study, for example, regarding such things as the distributions of types and timings of the malignancies among the different litters.

The authors also claimed that PPI altered cell populations in the haemopoietic system and induced cytogenetic damage; however, the magnitudes of those effects were slight in comparison with the extremely high induced mutation frequencies needed to explain their cancer results, as discussed above. Regarding the haemopoietic system, they concluded that PPI led to substantial alterations in certain cell populations, as exemplified by large increases in variability between animals but no changes in means. My main concern with this part of their paper relates to the normalizing procedure that was used to demonstrate increased variability in experimental groups. Examination of the summary data in Table 2 of their paper shows a high degree of variability between laboratories and strains. In view of the numerous significant differences, it is puzzling that the justification given by the authors for applying their normalizing procedure was 'to compensate for minor variations resulting from assays at different times, different venues and different mice' (p. 304). If the normalizing procedure was unjustified, their claimed effect may be no more than an artifact that tricked them into thinking that plutonium was inducing more variability. In view of the authors' conclusion, it also seems curious that the variances (as calculated from the summary data in their Table 2) of the experimental groups are not larger than those of the control groups, on average. It is interesting that ANOVA comparisons of individual dose groupings (with other variables held constant) showed that dose was often a significant factor, but responses were about as likely to go down, as up, with increasing dose.

Regarding the authors' conclusion that cytogenetic aberrations (mostly chromatid-type) resulted from PPI, I expect that important aberrations would show up in all cells containing chromosomes, regardless of tissue. They did not. In addition, there was a significant ($P < 0.05$) dose–response only in 2-day marrow. Because the aberration data also showed considerable variability, their interpretation regarding a possible effect of PPI demands considerable caution. In the face of so much variability in the cytogenetic and haemopoietic data, the authors simply provided too little information for anyone to evaluate carefully whether their data support their claims. That shortcoming becomes critical when one considers the implausibility that such small doses of PPI would influence numerous offspring in such a small study.

### ACKNOWLEDGEMENTS

The research was conducted at the Oak Ridge National Laboratory, managed by Lockheed Martin Energy Research Corp. for the US Department of Energy under contract number DE-AC05-960R22464. The US Government retains a non-exclusive, royalty-free licence to publish or reproduce the published form of the contribution, or allow others to do so, for US Government purposes.

*PB Selby*
*Life Sciences Division, Oak Ridge National Laboratory,*
*1060 Commerce Park Drive, Oak Ridge, TN 37830–6480, USA*

### REFERENCES

Lord BI, Woolford LB, Wang L, Stones VA, McDonald D, Lorimore SA, Papworth D, Wright EG and Scott D (1998) Tumour induction by methyl-nitroso-urea following preconceptional paternal contamination with plutonium-239. *Br J Cancer* **78**: 301–311

Lüning KG, Frolen H and Nilsson A (1976) Genetic effects of Pu-239 salt injections in male mice. *Mutat Res* **34**: 539–542

National Council on Radiation Protection and Measurements (1987) *NCRP Report No. 89*, NCRPM: Bethesda.

Selby PB (1990) Experimental induction of dominant mutations in mammals by ionizing radiations and chemicals. *Issues Rev Teratol* **5**: 181–253

UNSCEAR (United Nations Scientific Committee on the Effects of Atomic Radiation) (1993) Hereditary effects of radiation. In: *Sources and Effects of Ionizing Radiation*, pp. 729–804. United Nations: New York

### Reasons it is doubtful that preconceptional paternal irradiation with plutonium-239 had any effect on cancer induction by methyl-nitroso-urea—reply

**Sir**

Dr Selby doubts the concept or hypothesis that preconception paternal irradiation (PPI) can have any influence on patterns of malignancy in offspring of that irradiated father. He is not alone. In particular he concludes that our 'observed cancer differences (Lord et al, 1998*a*) have nothing to do with the injected plutonium'. It appears that he does, at least, accept that there were cancer differences. One should look first at what we did. We injected male mice with $^{239}$Pu and waited 12 weeks, thus ensuring continuous irradiation to, and throughout, an entire spermatogenic cycle, before mating with normal females. Their offspring were treated with methyl-nitroso-urea (MNU), which is known to induce thymic lymphoma and myeloid leukaemia. We observed acceleration and amplification of that process in the offspring of those mice injected with $^{239}$Pu compared to controls injected only with the carrier solution. Paternally injected plutonium was the only difference between the groups. We naturally concluded that $^{239}$Pu was the obvious link to the enhanced malignancy rate. We did not claim that it caused the malignancies. Indeed, we pointed out that without the MNU induction, no malignancies resulted. Neither, and most importantly, did we claim to understand the mechanisms. We simply reported the observations and threw out a few possible suggestions.

We would ask to take into account also a companion paper (Lord et al, 1998*b*), which Dr Selby has either missed or ignored and where we obtained similar results when using 3.3 Gy γ-rays as the secondary induction agent rather than MNU. In addition, we may now add that γ-ray PPI can be equally effective (KP Hoyes and BI Lord, to be published). Cumulatively, these experiments now represent groups approaching 200 mice in each arm of the study though even at the original 50 per group, the incidence statistics are more than favourable when compared with the size of

*© 1999 Cancer Research Campaign*

groups accepted in epidemiological studies. Furthermore, variation from replicate to replicate did not appear to be a problem. Our control groups matched exactly the cancer incidence reported 24 years earlier by Dexter et al (1974). We do not believe group size was a contributory factor in these observations. The statistics were analysed and the differences proved significant.

The main part of Dr Selby's argument lies in the expected potential mutation frequencies induced by $^{239}Pu$ and whether these match the *guestimated* frequencies he calculates from our observations. His estimates of radiation dose are probably reasonable. In response to questions we estimated 65 and 130 mGy over 3 months (Lord et al, 1998*b*). We assume Dr Selby's calculated doses are over 3 months also; he does not say. However, we have tended to play its significance down since it means so little. It is an average dose and takes no account of the inhomogeniety of dose distribution from an incorporated α-emitter and a single α-particle hit has been estimated to deliver about 0.5 Gy to, for example, stem cells in haemopoietic tissue (Lorimore et al, 1993). For equivalence in fertility effects we found it appropriate to deliver 3 Gy to the fathers in our new acute γ-irradiation studies. Dr Selby, therefore, is probably underestimating the radiation-induced mutation frequencies. He emphasizes that they must be of a specific type that *causes* leukaemia to develop. This is not the case. MNU causes the leukaemia to develop, probably in all the mice if thymomas did not develop first. PPI is only required to accelerate the development of leukaemia when triggered by MNU.

All Dr Selby's analysis is based on classical point mutation genetics. It is now clear that the phenomenon of genomic instability is a potential outcome of radiation exposure (see Mothersill, 1998); latent damage, which can be exposed after considerable delays and after completion of multiple cycles of apparently normal division with no mutations. It is not, therefore, necessarily detected by the classical methods. Much emphasis is now placed on potential 'bystander' effects (see Mothersill, 1998), which can amplify the frequency of these unstable mutations by virtue of unexpected interactions between irradiated and non-irradiated cells. This, in itself, would lead to different aberration rates in different tissues – a point highlighted by Dr Selby. There is evidence of epigenetic inheritance of defects in the germline and Cox (1992) described a number of ways in which genes may 'break the rules', concluding that 'It is now clear that non-Mendelian (epigenetic) processes operating during gamete formation can influence tumour susceptibility in man'.

Unlike in a court of law, where the recent case trying the validity of the PPI hypothesis was lost on 'the balance of probabilities', a scientific observation cannot be dismissed simply because it 'seems extraordinarily improbable'. A lack of understanding of the mechanisms does not, of itself, invalidate an observation.

*BI Lord*
*CRC Section of Haemopoietic Cell & Gene Therapeutic, Paterson Institute for Cancer Research, Christie Hospital NHS Trust, Manchester M20 4BX, UK*
*EG Wright*
*MRC Radiation and Genome Stability Unit, Chilton, Didcot, Oxon OX11 ORD, UK*

### REFERENCES

Cox R (1992) Transgeneration carcinogenesis: are there genes that break the rules? *Radiat Protect Bull* **129**: 15–23

Dexter TM, Schofield R, Lajtha LG and Moore M (1974) Studies on the mechanisms of chemical leukaemogenesis. *Br J Cancer* **40**: 325–331

Lord BI, Woolford LB, Wang L, Stones GA, McDonald D, Lorimore SA, Papworth D, Wright EG and Scott D (1998*a*) Tumour induction by methyl nitroso urea following preconceptional paternal irradiation with plutonium-239. *Br J Cancer* **78**: 301–311

Lord BI, Woolford LB, Wang L, Stones VA, McDonald D, Lorimore SA, Wright EG and Scott D (1998*b*) Induction of lympho-haemopoietic malignancy: impact of preconception paternal irradiation. *Int J Radiat Biol* **74**: 721–728

Lorimore SA, Goohdead DT and Wright EG (1993) Interaction of haemopoietic stem cells by slow α-particles. *Int J Radiat Biol* **63**: 655–660

Mothersill C (ed) (1998) State-of-the-art report of current research on 'Genomic Instability'. *Int J Radiat Biol* **74**: 663–804

 *© 1999 Cancer Research Campaign*

